# Amyloid *β* Enhances Typical Rodent Behavior While It Impairs Contextual Memory Consolidation

**DOI:** 10.1155/2015/526912

**Published:** 2015-07-01

**Authors:** Karla Salgado-Puga, Roberto A. Prado-Alcalá, Fernando Peña-Ortega

**Affiliations:** ^1^Departamento de Neurobiología del Desarrollo y Neurofisiología, Instituto de Neurobiología, Universidad Nacional Autónoma de México, 76230 Juriquilla, Querétaro, QRO, Mexico; ^2^Departamento de Neurobiología Conductual y Cognitiva, Instituto de Neurobiología, Universidad Nacional Autónoma de México, 76230 Juriquilla, Querétaro, QRO, Mexico

## Abstract

Alzheimer's disease (AD) is associated with an early hippocampal dysfunction, which is likely induced by an increase in soluble amyloid beta peptide (A*β*). This hippocampal failure contributes to the initial memory deficits observed both in patients and in AD animal models and possibly to the deterioration in activities of daily living (ADL). One typical rodent behavior that has been proposed as a hippocampus-dependent assessment model of ADL in mice and rats is burrowing. Despite the fact that AD transgenic mice show some evidence of reduced burrowing, it has not been yet determined whether or not A*β* can affect this typical rodent behavior and whether this alteration correlates with the well-known A*β*-induced memory impairment. Thus, the purpose of this study was to test whether or not A*β* affects burrowing while inducing hippocampus-dependent memory impairment. Surprisingly, our results show that intrahippocampal application of A*β* increases burrowing while inducing memory impairment. We consider that this A*β*-induced increase in burrowing might be associated with a mild anxiety state, which was revealed by increased freezing behavior in the open field, and conclude that A*β*-induced hippocampal dysfunction is reflected in the impairment of ADL and memory, through mechanisms yet to be determined.

## 1. Introduction

Alzheimer's disease (AD) is a disorder characterized by severe cognitive impairments [1–3] and by the presence of senile plaques that contain the amyloid beta peptide (A*β*) [3–5]. A strong correlation between the levels of soluble oligomeric forms of A*β* and the cognitive decline in AD patients [3–5] has been further supported by the findings that intracerebral infusion of A*β*, particularly into the hippocampus, disrupts learning and memory in rodents [6–13].

Earlier in AD, deterioration of hippocampal function, likely induced by soluble A*β*, contributes to the initial memory deficits observed in patients [3, 14]. This observation has been reproduced in transgenic animal models of AD [15–17]. Interestingly, AD is also related to the deterioration in activities of daily living (ADL) [18], which has been partially associated with changes in hippocampal function measured both behaviorally [15, 16] and electrophysiologically [19]. Burrowing is a typical rodent behavior that has been proposed as a hippocampus-dependent assessment of ADL in rodents [18, 20, 21] and/or as an assessment of proper hippocampal function [22]. Although burrowing is reduced in AD transgenic mice [15, 16, 23], it has not yet been investigated whether this reduction is due to A*β* and if it parallels the well-known A*β*-induced memory impairment. Thus, the purpose of this study is to test whether or not A*β* affects burrowing at doses that induce hippocampus-dependent memory impairment. Surprisingly, our results show that intrahippocampal application of A*β* increases burrowing while inducing memory impairment. We consider that this A*β*-induced increase in burrowing should not be interpreted as an improvement in hippocampal function or the animal's well-being, but it should rather be associated with a disruption of the emotional (anxiety and/or alertness) state of the animals, as has been demonstrated in other experimental situations in which burrowing is pathologically increased [24–29]. This possibility is supported by our observation that A*β* increases freezing behavior while the animals are exposed to an open field.

## 2. Materials and Methods

### 2.1. Ethics Statement

All experimental procedures were approved by the Bioethics Committee of the Instituto de Neurobiología, UNAM and were carried out according to the guidelines of the Institutional Animal Care and Use Committee Guidebook (NIH publication 80-23, Bethesda, MD, USA, 1996).

### 2.2. Subjects

Adult male* Wistar* rats (300–330 g) from the breeding colony at our Institute were housed individually in transparent acrylic cages in a temperature-controlled* vivarium* (22 ± 1°C) and maintained under a 12 h light: 12 h dark cycle (lights on at 7:00 a.m.) with food and water* ad libitum*. They were kept in these conditions for seven days before the experiments started and throughout this study.

### 2.3. Surgical Procedure

Animals were anesthetized with sodium pentobarbital (62 mg/kg, i.p.), followed by the injection of atropine sulfate (1 mg/kg, i.p.), and positioned in the stereotaxic instrument (Stoelting Co. IL). Stainless steel guide cannulae (23-gauge, 10 mm long) were bilaterally implanted into the dorsal hippocampus CA1 region (AP = −4.0, *L* = ±3.0, *V* = −2.55) [30]. The cannulae were affixed to the skull using two screws and dental acrylic, and a stylet was inserted in each cannula and maintained there at all times except during microinjection. The animals were allowed 7 days to recover from the surgical procedure before drug administration. During this time, the animals were gently handled (3–5 min) on three separate days.

### 2.4. Amyloid Beta Preparation

A*β* was obtained from Bachem (Heidelberg, Germany) and oligomerized as previously described [31, 32]. Briefly, 1,1,1,3,3,3-hexafluoro-2-propanol (HFIP) was added to solid A*β*_1–42_ to make a solution with a final peptide concentration of 1 mM. Then, it was incubated for 60 min at room temperature. HFIP was evaporated overnight, and a 5 mM solution of A*β*_1–42_ was prepared by adding DMSO. This solution was diluted with F12 medium (MF12) to 100 pmoles A*β*_1–42_/*μ*L. Then, this solution was incubated 24 h at 5°C. Subsequently, the solution was centrifuged at 14,000 ×g for 10 min at 4°C. Finally, A*β* oligomers contained in the supernatant were collected and used for the experiments. Previous characterization of this preparation showed the presence of A*β* monomers, oligomers, and some protofibrils [32].

### 2.5. Drug Administration

All animals were bilaterally infused with vehicle (MF12) or A*β* (100, 200, or 400 pmoles/side) into the CA1 region of the hippocampus, a total of 200 (1 *μ*L/side), 400 (2 *μ*L/side), or 800 (4 *μ*L/side) pmoles per animal, respectively. The infusion was made with a 30-gauge injection needle (11 mm long) connected to a Hamilton microsyringe by polyethylene tubing. The infusion rate (0.2 *μ*L/min) was controlled by a microinfusion pump (WPI, 220i). After the infusion, the injection needles remained for 5 min inside the guide cannulae to allow proper drug diffusion. Behavioral tests started three weeks after drug administration.

### 2.6. Behavioral Tests

#### 2.6.1. Typical Behavior Test

Evaluation of typical, hippocampus-dependent behavior was made using the “*burrowing*” task [22, 33]. Three hours before the start of the dark cycle (4:00 pm), the animals were placed in a cage (40 × 30 × 30 cm) containing a “burrow,” that is, a black plastic tube (30 cm long and 10 cm in diameter) filled with clay balls (1540 g). Food and water were provided* ad libitum*. After 2 h and 18 h, the clay balls removed from the burrow were weighed (weight burrowed).

#### 2.6.2. Motor Activity Test

To evaluate motor activity, animals were placed in a cage (40 × 20 × 50 cm) containing a running wheel. All the animals were allowed to use the running wheel freely for two hours (4:00–6:00 pm). Parameters of distance and velocity were recorded with the Activity Wheel Monitor (AWM) software (Lafayette Instruments, version 11.12).

#### 2.6.3. Open Field Test

To evaluate their anxiety level, animals were placed in one of the four corners of an open field arena (70 cm × 50 cm × 30 cm, divided into 10 × 10 cm^2^ squares) and allowed to explore it for 5 minutes, while videotaped to assess, off-line, the distance traversed, the time spent in the arena's center, the amount of rearing, the time spent grooming or freezing, and the defecation frequency [34].

#### 2.6.4. Contextual Memory Test

Animals were trained in a single-trial, step-through, inhibitory avoidance task, as described in detail elsewhere [35]. Briefly, the training apparatus was divided into two compartments (30 × 30 × 30 cm each), separated by a guillotine door. The safe compartment has a floor of stainless steel bars (6 mm in diameter, separated by 1.5 cm) and a 10-Watt light bulb located in the center of its lid. The darker V-shaped shock compartment has walls and floor made of stainless steel plates, 20 cm wide at the top and 8 cm wide at the bottom, where a 1.5 cm slot separates the two stainless steel plates. These plates could be electrified using a square-pulse stimulator (Grass S-48) in series with a constant current unit (Grass CCU-1A). The training apparatus was located inside a dark, sound-proof room provided with background masking noise. For training, the animals were placed in the safe compartment and 10 s later, the guillotine door was opened and the latency to enter the shock compartment was recorded (training latency). Once the animal was completely inside the dark chamber, the door was closed, and a foot-shock (0.7 mA) was delivered. After 5 s, the door was opened, allowing the animal to escape into the safe compartment (escape latency). Once the animal was in the safe compartment, the door was closed; the animal was left there for 30 s and then put back in its home cage. Memory evaluation (retention test) was performed 48 h later, and the latency to enter the shock compartment was measured (retention latency). The test was ended either when the animal entered the dark compartment or after 600 s without entry, and a score of 600 was assigned. If the animal entered the dark compartment the foot-shock was not delivered.

### 2.7. Histology

In order to verify the location of cannulae tips, all the animals were sacrificed with an overdose of sodium pentobarbital and perfused transcardially with isotonic saline and 10% formaldehyde. The brain was removed and fixed in 10% formaldehyde for 6 days. Then, sagittal hippocampal slices (10–50 *μ*m) were obtained using a cryostat (Leica CM 1850) and stained with toluidine blue [36, 37]. The sections were examined under a light microscope for two purposes, to determine the location of the injection needle tips and to assess for the integrity of the hippocampal formation. The integrity of the hippocampal pyramidal and granular fields was assessed using light microscopy. Micrographs obtained at 4x magnification were used to quantify the area of such fields with an image analyzer system (NIH Image J 1.47) [36–38].

### 2.8. Statistics

Only animals with both cannulae located in the hippocampal CA1 region were included in the statistical analyses. Typical behavior and learning and memory scores were analyzed with nonparametric statistics. Independent Kruskal-Wallis analyses of variance were computed to compare weight burrowed and training, escape, and retention latencies among groups. To make comparisons within and between groups, the Wilcoxon signed-rank test and the Mann-Whitney *U*-test were used, respectively. Data are presented as median ± interquartile ranges.

Motor activity in the running wheel and all measurements in the open field (distance traversed, time in the arena's center, amount of rearing, duration of grooming and freezing, and defecation frequency), as well as hippocampal area measurements, were analyzed with the Student's *t*-test for independent samples, and the data are presented as mean ± S.E.M. Correlations were made with the nonparametric Spearman correlation test and linear regression analysis. To obtain each animal's weight gain, its weight on the day of surgery (set as 100%) was compared with its weight right before behavioral testing. Weight gain between groups was analyzed with the one-way ANOVA test. For graphs and statistical analysis, the Prism Graph Pad software (version 5.0) was used.

## 3. Results

### 3.1. Intrahippocampal A*β* Injection Affects Contextual Memory

In order to determine the doses of A*β* that induce contextual memory deficits, the CA1 area of the hippocampus was bilaterally microinjected either with vehicle (MF12, control group) or with 200, 400, or 800 pmoles/rat of A*β*.

To evaluate possible changes in food intake induced by A*β* treatment, each rat's weight was measured on the day of surgery and again right before behavioral tests. Intrahippocampal injection of 200, 400, or 800 pmoles of A*β* did not significantly alter the weight gain of the subjects (114 ± 2.9%, 113.5 ± 2.5%, and 121.0 ± 3.3%, resp.) compared to the control group (114 ± 3.5%; *F*(3) = 0.93, *p* > 0.4).

**Figure 1:**
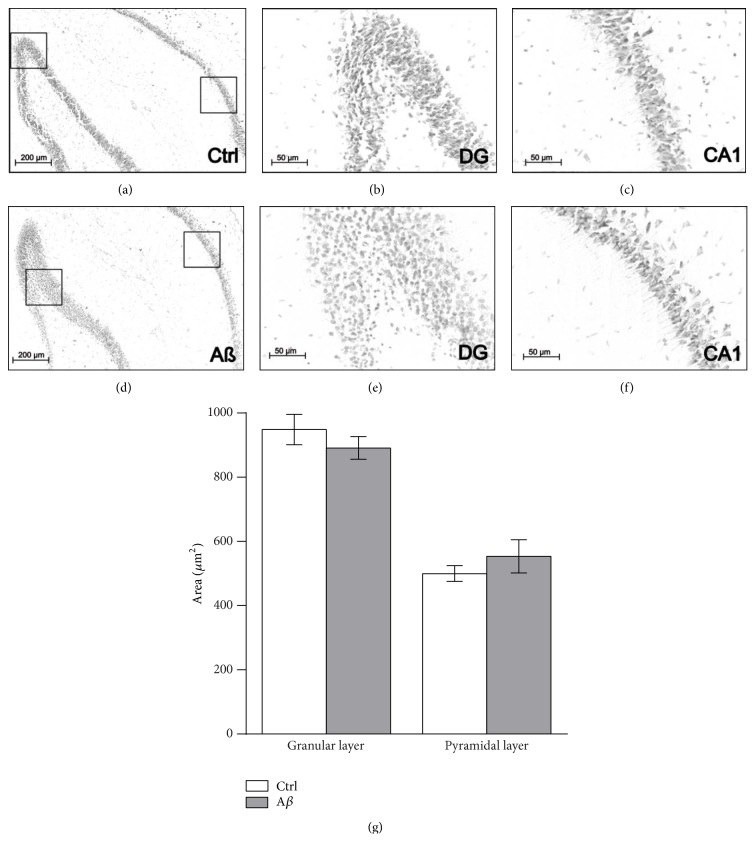
A*β* does not affect hippocampal integrity. ((a)–(f)) Representative microphotographs of hippocampal sections obtained from animals injected with vehicle ((a)–(c)) or 400 pmoles of A*β* ((d)–(f)). ((a) and (d)) Panoramic view of the hippocampus (10x). ((b) and (e)) Magnification of the dentate gyrus (40x) from the area enclosed in the squares shown in (a) and (d), respectively. ((c) and (f)) Magnification of the CA1 region (40x) from the area enclosed in the squares shown in (a) and (d), respectively. (g) Quantification of the area (mean ± S.E.M) of the granular cell layer and the pyramidal cell layer from sections of animals injected with vehicle (Ctrl) or 400 pmoles of A*β* (*n* = 10/group). Note that A*β* injection does not modify hippocampal integrity measured as the granular or pyramidal area.

To evaluate possible changes in hippocampal integrity induced by A*β* treatment, histological sections of animals treated with either MF12 or with 400 pmoles of A*β* were stained and evaluated using light microscopy (Figures 1(a)–1(f)). The quantification of the area of the granular and pyramidal layers shows no significant differences between groups (*p* > 0.3). These results indicate that the A*β* does not affect hippocampal integrity at the time and the dose tested.

**Figure 2:**
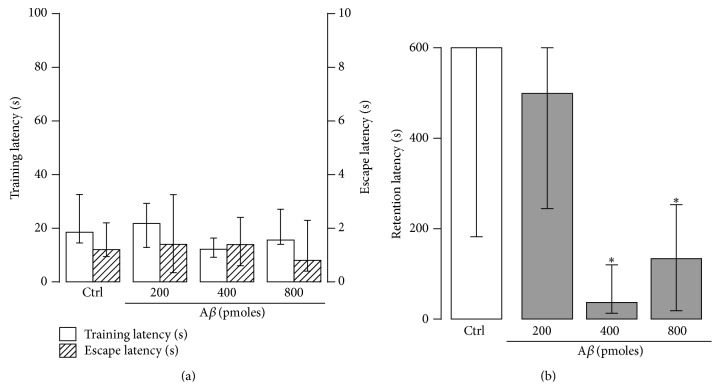
A*β* induces contextual memory deficits. (a) Training and escape latencies of groups injected into the hippocampus with MF12 (Ctrl) or A*β* (200, 400, and 800 pmoles) and then evaluated in the inhibitory avoidance task. There were no significant differences among the groups in either latency. (b) Retention latencies of Ctrl and A*β* groups measured 48 h after training (*n* = 6–11 rats/group). Data are presented as medians ± interquartile ranges. ^*∗*^ *p* < 0.05 versus Ctrl.

Contextual memory was evaluated in the step-through inhibitory avoidance task. There were no significant differences in training and escape latencies among the control group and the A*β* groups (Figure 2(a), *H*(3) = 5.6, *p* > 0.1 and *H*(3) = 1.1, *p* > 0.8) suggesting that neither the motor capabilities necessary to perform the task, nor the detection of the foot-shock was impaired by A*β*. Thus, the inference can be made that the drug did not hinder the afferent and efferent processes necessary to perceive and react to the aversive stimulation.

In contrast, some A*β* treatments did change retention latencies of the subjects (Figure 2(b), *H*(3) = 18.2, *p* < 0.0005). In particular, injection of 400 and 800 pmoles of A*β* produced a significant impairment in the retention latency compared to the control (*p* < 0.01 in both cases, Figure 2(b)). However, injection of 200 pmoles of A*β* did not alter retention scores (*p* > 0.4 versus Ctrl). Moreover, pooled scores of subjects with injection outside the hippocampal formation showed a normal retention score (*m*_*e*_ = 600.0 s, data not shown). Together, these results show that a single bilateral injection of 400 or 800 pmoles of A*β* in the hippocampal CA1 region induces a deficit in long-term contextual memory.

**Figure 3:**
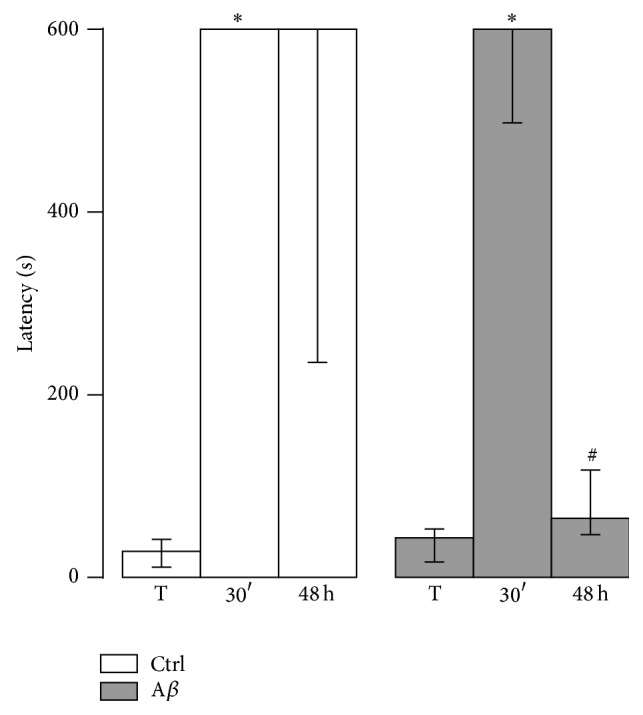
A*β* impairs memory consolidation but not learning. Training latencies (T) and retention latencies obtained 30 min (30′) and 48 h after training from control (Ctrl) or A*β* (400 pmoles) groups (*n* = 10 rats/group). Data are presented as medians ± interquartile ranges. ^*∗*^ *p* < 0.05 versus training latency. ^#^ *p* < 0.05 versus 30 min retention latency.

To discern whether the performance deficit produced by A*β* was due to disruption of either learning or memory consolidation, we evaluated the performance of the avoidance task at 30 min and at 48 h after training in two groups of rats. One group was treated with MF12 and the other with 400 pmoles of A*β*. Both groups showed perfect retention scores in the 30 min retention test (*p* < 0.05, Wilcoxon test, as compared to the corresponding control group) while a significant retention deficit was evident in the A*β* group only in the 48 h retention test, which showed a significantly lower retention latency than in the 30 min retention test (*p* < 0.005, Wilcoxon test) (Figure 3). The idea that the amnesic effect of contextual memory was due to an impediment of consolidation and not of learning was confirmed by the fact that in spite of the retention deficit shown in the 48 h test, treated animals showed excellent short-term memory scores when tested 30 min after training, which indicates that they had learned the conditioned response.

### 3.2. Intrahippocampal A*β* Injection Increases Burrowing

**Figure 4:**
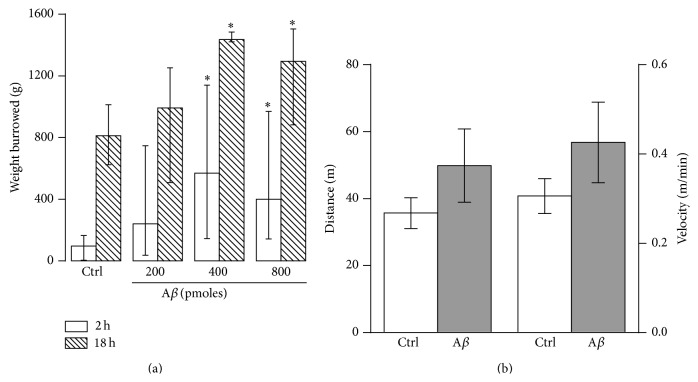
A*β* increases burrowing without affecting motor activity. (a) Weight burrowed in the 2 h and 18 h burrowing tests by the control (Ctrl) and the groups treated with 200, 400, and 800 pmoles A*β* (*n* = 6–11 rats/group). Data are presented as medians ± interquartile ranges. ^*∗*^ *p* < 0.05 versus Ctrl. (b) Distance (left axis) and velocity (right axis) displayed by the control (Ctrl) and the A*β* (400 pmoles) groups during the 2 h of motor activity on the free running wheel (*n* = 9–11 rats/group). Data are presented as means ± S.E.M.

To evaluate the effect of A*β* injection on a typical, hippocampus-dependent behavior, the subjects were microinjected with vehicle (MF12) or with 200, 400, or 800 pmoles of A*β* and tested in the burrowing task (Figure 4). Analysis of the weight burrowed in the first 2 h of the task shows significant differences among the groups (*H*(3) = 10.5, *p* < 0.05). A*β* treatment with 400 and 800 pmoles induced a significant increase compared with the control group (*p* < 0.01, Figure 4(a)). In contrast, injection of 200 pmoles of A*β* did not significantly increase the weight burrowed (*m*_*e*_ = 240.0 g, *p* > 0.1).

The A*β*-induced increase in burrowing was also observed at 18 h. Again, the injection of 400 and 800 pmoles of A*β* (*m*_*e*_ = 1435.0 g and *m*_*e*_ = 1295.0 g, resp.), but not of 200 pmoles of A*β* (*m*_*e*_ = 992.0 g, *p* > 0.3), induced a significant increase of the weight burrowed (*p* < 0.05) compared with the control group (*m*_*e*_ = 812.5 g) (Figure 4(a)).

To assess if the A*β*-induced increase in the burrowing behavior was due to a change in motor activity, such as hyperactivity, independent groups treated with either MF12 or with 400 pmoles of A*β* were tested on the running wheel for 2 h (Figure 4(b)). Distance and velocity of free running were measured. The injection of 400 pmoles of A*β* did not significantly modify the distance or the velocity, as compared with the control group (*p* > 0.1). Together, these results show that the A*β*-induced increase in burrowing is not related to a change in spontaneous motor activity (Figure 4(b); also, see Figure 6(a)).

### 3.3. A*β*-Induced Memory Deficits Correlate with the Enhanced Burrowing

**Figure 5:**
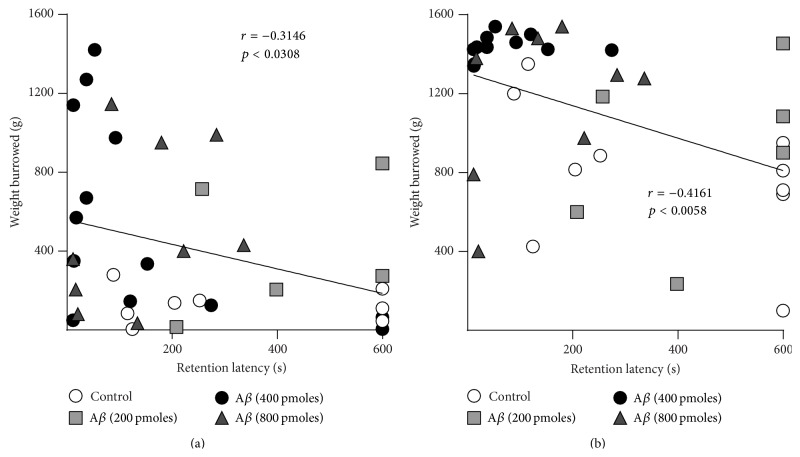
Correlation between A*β* impairment in contextual memory and the increase in burrowing behavior. The median of the weight burrowed during 2 h (a) or 18 h (b) is plotted against the median of the retention latency score from each animal treated with MF12 (control) or 200, 400, and 800 pmoles of A*β* (*n* = 6–11 rats/group).

Since the injection of 400 and 800 pmoles of A*β* induces a significant increase in burrowing behavior and the same doses produce a significant reduction in memory, we performed a correlation analysis in order to determine a possible correlation between these two A*β*-induced alterations (Figure 5). Correlation analysis of retention latencies and weight burrowed at 2 h (Figure 5(a)) or at 18 h (Figure 5(b)) showed a significant negative correlation (*p* < 0.05). Together, these results suggest that A*β* produces hippocampal dysfunction that is reflected in both a memory deficit and an increase in burrowing.

**Figure 6:**
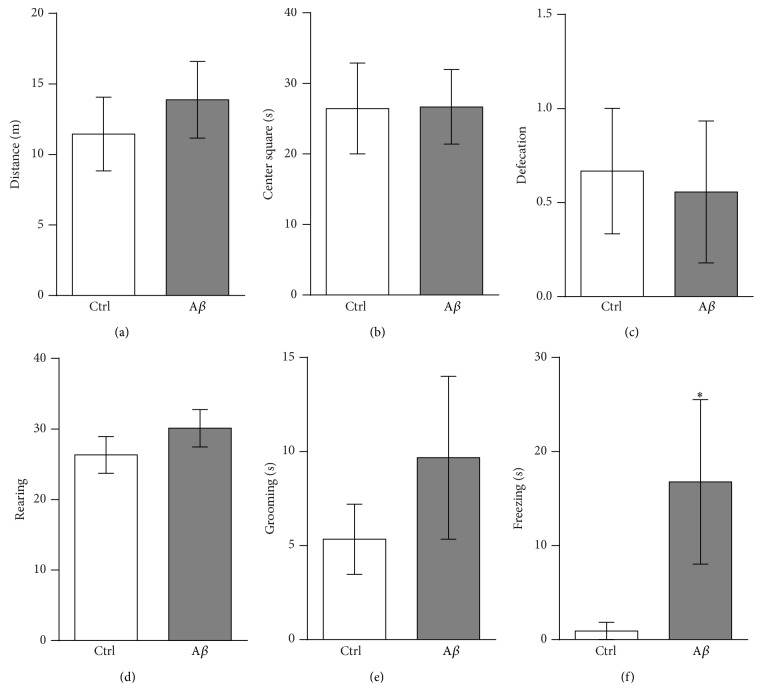
A*β* induces mild anxiety. (a)–(f) Quantifications of anxiety-related behaviors evaluated during the 5 min test in the open field arena. Such measurements included (a) the distance traversed; (b) the time spent in the arena's center; (c) the defecation frequency; (d) the amount of rearing; (e) the time spent grooming and (f) freezing. Note that the only parameter that significantly increased in A*β*-treated animals is the duration of the freezing behavior. Data are presented as means ± S.E.M. ^*∗*^ *p* < 0.05 versus Ctrl.

Since an increase in burrowing has been associated with anxiety [27, 29], independent groups treated with either MF12 or with 400 pmoles of A*β* were tested in the open field [34]. We found no significant differences between them in most of the anxiety-related parameters measured during 5 minutes in the open field test (Figures 6(a)–6(e)) [34]. However, A*β*-treated animals spent more time frozen (16.78 ± 8.74 s) compared to control animals (0.92 ± 0.92 s) (*p* < 0.05; Figure 6(f)). These results indicate that A*β*-induced increase in burrowing might be related to a mild increase in anxiety, which is not strong enough to be reflected neither in other parameters measured in the open field (Figures 6(a)–6(e)) nor in the training and escape latencies of the inhibitory avoidance test (Figure 2(a)).

## 4. Discussion

Here, we show that a single intrahippocampal application of A*β* induces an impairment of memory, but not of learning, as well as increased burrowing and freezing behaviors without affecting locomotion or hippocampal integrity. Our result that A*β* affects contextual memory is consistent with the overwhelming evidence that intracerebroventricular or intrahippocampal application of A*β* induces a deficit in retention of the inhibitory avoidance task [6–13, 39–41].

There is still some controversy regarding the effect of A*β* on the learning phase of this task. Most studies, including ours, have shown that the A*β*-induced memory impairment in the inhibitory avoidance test is not related to a learning impairment [6–10, 12, 13, 39, 40]; however, some studies have reported that A*β* affects both learning and memory in this task [11, 41]. It is likely that these differences can be explained by minor, but important, methodological differences [42]. In the study by Garcia-Osta and Alberini [41], A*β* was acutely applied during different phases of the task, whereas we applied A*β* three weeks before the test. Thus, it is possible that the acute and chronic effects of A*β* can differentially alter learning. As another example, Jiang et al. [11] used the short A*β* sequence (A*β*_25–35_), which is known to produce different effects on hippocampal function from those produced by full-length A*β* [43–47].

Thus, in view of the vast evidence that A*β* induces memory impairment without affecting learning in the inhibitory avoidance task [6–10, 12, 13, 39, 40], we favor the notion that A*β* hinders the performance of the animals in this memory test by affecting memory consolidation.

Our finding that A*β* increases burrowing is more difficult to explain. First of all, our data might appear to contradict the evidence provided by others that burrowing is reduced, along with memory performance, in two different AD transgenic mice [15, 16, 23]. Note, however, that the decrease in burrowing observed in AD transgenic mice is quite erratic, since at 1–3 months of age transgenic animals exhibit either a reduction [15] or no change in this behavior [23], and, again at 9-months of age, they show either a reduction [23] or no change in burrowing [15]. Furthermore, Deacon et al. [15] observed that the reduction in memory performance in Tg2576 mice does not necessarily parallel the changes in burrowing. Thus, our experimental conditions (single application of A*β*) might be producing a different pathological state than the one found in AD transgenic mice, at least with respect to the combination of memory and burrowing alterations. Along these lines, Deacon et al. [15] showed that the changes in burrowing are not age-dependent, whereas previous experiments from our laboratory have shown that the deleterious effects of A*β* on different brain circuits, including the hippocampus, are indeed age-dependent [32, 48].

There are many possible explanations for the A*β*-induced increased burrowing observed in this study. The natural tendency of rodents to burrow is a highly conserved behavior [16, 21, 49, 50] that has been used to assess hippocampal functioning [21, 22, 51] and animal's well-being [21, 50, 52]. Burrowing behavior is decreased by hippocampal lesions or dysfunction [21, 22, 51] and by pathological conditions such as chemotherapy-induced mucositis [52], inflammation [50, 53–55], stress [51], pain [56, 57], a high-fat diet [58], anxiety [25], prion infection [20, 59], and after laparotomy [60]. So far, we have no clear indication that the A*β*-induced increased burrowing is due to an improvement of the animal's well-being or of hippocampal function. On the contrary, we have shown that intracerebroventricular application of A*β* indeed reduces hippocampal network function [38, 61].

Since burrowing is utilized in nature to hide from predators and to conceal food [49], it has also been associated with anxiety [58], and it has even been catalogued as a depressive/anxiety-like behavior [58]. Supporting this possibility, there is evidence that burrowing can be increased by depressive/anxiety-promoting conditions such as fasting [58], neonatal isolation [28], high-fat diet [29], and grid floor housing [27]. In the latter two cases, the increase in burrowing has been associated with clear anxiety signs [27, 29]. Furthermore anxiolytic drugs such as pregabalin [53] and gabapentin [54] reduce burrowing. However, one report has shown a strong correlation between anxiety signs and decreased burrowing induced by stress [51].

Despite some evidence that A*β* induces anxiety [7, 62], we cannot associate the A*β*-induced increase in burrowing observed in this study to an excessive anxiety state of the animals, since a presumptive anxiety state in our A*β*-treated animals was not reflected as a change in the training and escape latencies of the inhibitory avoidance test (Figure 2) as a change in locomotion (Figure 4) or as changes in the distance traversed, the time in the arena's center, the amount of rearing, the time in grooming, or the defecation frequency in the open field test (Figure 6). We only found a significant increase in the time that A*β*-treated animals spent frozen in the open field (Figure 6(f)). In contrast, there is evidence that animals with reduced anxiety, such as the 5-HT transporter overexpressing mice [25] or aged senescence-accelerated prone mouse 8 (SAMP; P8) [63] exhibit increased burrowing. At this point, we have no conclusive evidence to exclude or accept the participation of anxiety in the A*β*-induced increase of burrowing observed in our experimental conditions. It is likely that A*β* induced a slight anxiety state in the animals that was revealed by the increased burrowing and freezing behavior in the open field, but it could not be properly quantified with our other measurements (motility, training, and escape latencies, and most of the measurements in the open field).

Alternatively, it has been proposed that burrowing may represent a reward/pleasure behavior. Supporting this possibility, Sherwin et al. [64] demonstrated that mice can be motivated to burrow and can be trained to press a lever to access burrowing material, even when there is no immediate need to burrow. Additionally, dopamine antagonism, which interferes with reward, reduces burrowing in mice [65]. Therefore, another possibility is that the A*β*-induced increase of burrowing observed in our experimental conditions may reflect a reward-seeking behavior. It has been suggested that voluntary wheel running can be considered as a rewarding behavior [66, 67] but, in our experimental conditions, A*β*-treated animals did not show any difference in wheel running compared to control animals (Figure 3). The latter observation, which is in agreement with evidence that A*β* does not affect locomotion [7, 8, 10, 12, 41], also excludes the possibility that increased burrowing is produced by a hyperactive state in our A*β*-treated animals, as could be suggested by the hyperactivity observed consistently in AD transgenic mice [68–70].

Another possible explanation for the increase in burrowing observed in our A*β*-treated animals is that such behavior is reflecting a negative change in the general emotional state of the animals. This possibility is supported by the fact that a consistent increase in burrowing has been observed in animals upon withdrawal from morphine [71–75], codeine [71], meperidine [71], and methadone [76, 77]. In these cases, burrowing has been considered as an “escape digging” behavior. This possibility is further supported by the evidence that animals increase burrowing when exposed to the predator fox odor, 2,5-dihydro-2,4,5-trimethylthiazoline [24], or synthetic pyrethroid (cyfluthrin) [26]. So far, the evidence discussed here points to the conclusion that the A*β*-induced increase in burrowing may reflect a disturbance in the emotional state of the animals (perhaps mild anxiety, escape digging, or both) rather than an improvement in hippocampal function or well-being because there is a clear A*β*-induced disturbance in hippocampal network activities (for a review, see [42]).

Both inhibitory avoidance [35, 78–81] and burrowing tests [21, 22, 51] are dependent on hippocampal integrity and function. The finding that both behaviors are affected by A*β*, without evidence of hippocampal damage (Figure 1), is consistent with previous observations that A*β* inhibits hippocampal network activity both* in vitro* ([38, 43, 46, 61, 82], for a review, see [19]) and* in vivo* ([38, 46, 61, 62, 83, 84], for a review, see [19]). Thus, we hypothesize that both the A*β*-induced increase in burrowing and the alteration in memory observed in this study are behavioral manifestations of hippocampal network disruption [38, 61, 62] rather than hippocampal damage. Therefore, we consider that exploring strategies to restore normal hippocampal network function would be beneficial against the behavioral alterations observed in A*β*-treated animals and perhaps in AD patients.
